# The time for surgery of peritonitis associated with peritoneal dialysis

**Published:** 2016

**Authors:** O Mihalache, C Bugă, H Doran, E Catrina, F Bobircă, A Andreescu, P Mustățea, T Pătrașcu

**Affiliations:** * ”Carol Davila” University of Medicine and Pharmacy, Bucharest, Romania; ** ”I. Juvara” Surgical Clinic, ”Dr. I. Cantacuzino” Clinical Hospital, Bucharest, Romania

**Keywords:** peritonitis, peritoneal dialysis, refractory peritonitis

## Abstract

Peritonitis is the main complication of peritoneal dialysis (PD) and also an important factor for raising the cost of the method to the level of hemodialysis. Associated with PD, peritonitis is responsible for the increase of morbidity and mortality of the procedure and, at the same time, the main cause of the technique failure. Severe and prolonged peritonitis or repeated episodes of peritonitis lead to ultrafiltration failure. Peritonitis treatment should aim for a rapid remission of inflammation in order to preserve the peritoneal membrane functional integrity. The treatment of PD peritonitis consists mainly of antibiotic therapy, surgical intervention not being usually required. However, it is of outmost importance to differentiate the so-called “catheter related” peritonitis from secondary peritonitis due to visceral lesions, in which the surgical treatment comes first. The confusion between secondary and “catheter related” peritonitis may lead to serious errors in choosing the correct treatment, endangering the patient’s life.

The differential diagnosis between a refractory or secondary peritonitis in a peritoneal dialyzed patient may be very difficult. In front of a refractory PD peritonitis, surgical exploration must not be delayed. Also we have to keep in mind that the aim of peritonitis treatment is the saving of the peritoneal membrane and not the catheter.

## Overview

Peritonitis represents the main complication of peritoneal dialysis (PD), being also responsible for rising the costs of this therapy to the level of hemodialysis. Associated with PD, peritonitis is the leading cause of the method failure, being responsible for rising its morbidity and mortality. Repeated episodes of peritonitis lead to the loss of ultrafiltration capacity of the peritoneal membrane [**1**].

Despite the number of peritonitis episodes dropping from about 6 episodes/year/patient in 1980 to 0,35 episodes/year/patient, due to the development of peritoneal dialysis systems, peritonitis remains the main problem of this method [**2**]. 

The etiological diagnosis of PD related peritonitis has to be well sustained by meticulous multidisciplinary assessment. The conservative treatment in secondary peritonitis could delay the correct surgical approach and may lead to the loss of the patient’s life.

## Cases presentation

Two representative cases of peritonitis associated with PD were presented by illustrating the difficulty of the differential diagnosis between secondary peritonitis and refractory “catheter related” peritonitis.

**Fig. 1:**
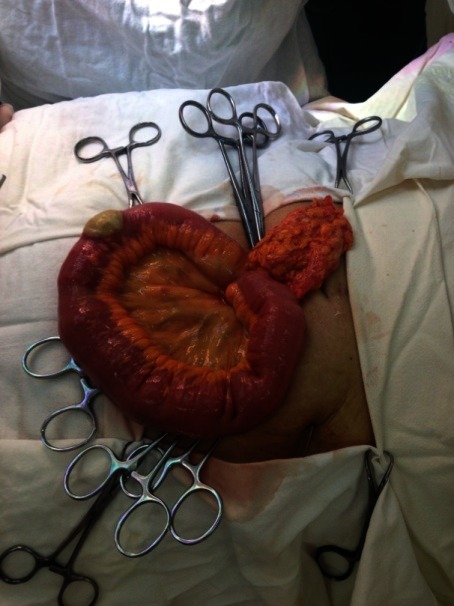
Intraoperative aspect - necrosis parcelar of the ileum

**Fig. 2:**
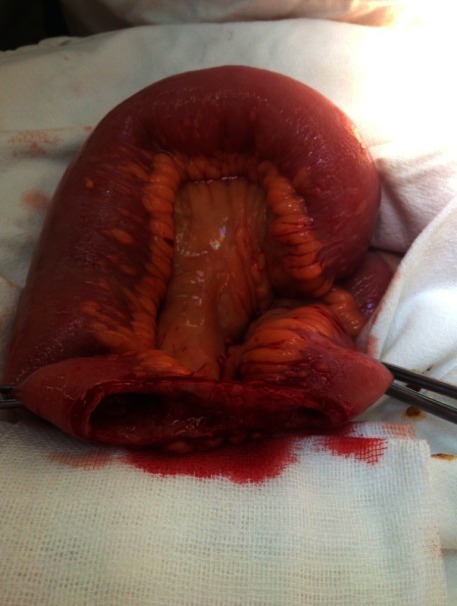
Intraoperative aspect after resection of the necrosis - intestinal mucosa with ischemic appearance, which needed segmental resection

**Fig. 3:**
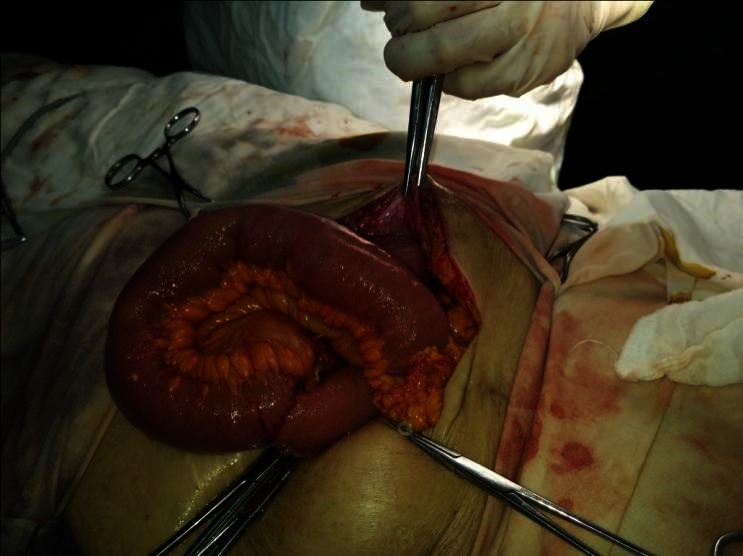
Intraoperative aspect - end-to-end anastomosis

The first case was a female patient, aged 77, with diabetic renal disease. Peritoneal dialysis was initiated 2 months prior to the actual episode by laparoscopic catheter insertion. She was admitted in the nephrology department for a neuro-psychiatric disorder, which led to the incapacity of performing the PD exchange. During hospitalization, she developed peritonitis with enterococci refractory to antibiotic treatment. The indication for catheter removal was established and the patient referred to our clinic. No abdominal sings of peritoneal irritation were noticed at presentation. The exploration of the peritoneal cavity during the catheter removal procedure revealed a necrosis parcelar on the antimesenteric border of the terminal ileum (**Fig. 1**). A segmental enterectomy with end to end anastomosis was performed (**Fig. 2**,**3**), the catheter was removed, and the peritoneal cavity was drained. 

Regarding this case, we had to underline the dimmed symptomatology and the low intensity pain decreasing from previous days. The unusual pathologic germ found and the persistence of the cloudy effluent despite the standard intraperitoneal antibiotic therapy represented the reasons that determined us to perform a comprehensive exploration of the peritoneal cavity. The real cause of peritonitis was determined intraoperatively.

**Fig. 4:**
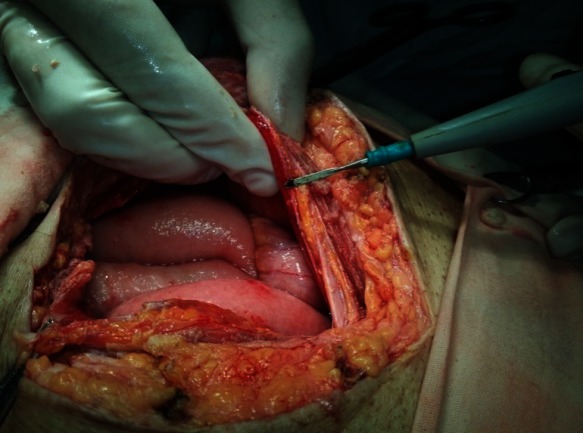
PD peritonitis - false membrane

**Fig. 5:**
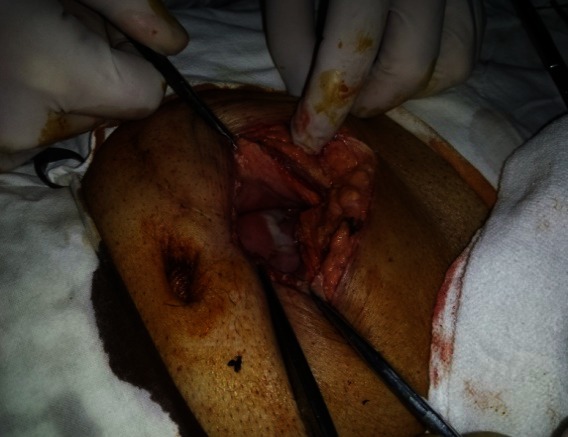
Thickened parietal peritoneum

The second case was a 69-year-old man, 7 months under PD, referred for a staphylococcus aureus refractory peritonitis without remission after 6 days of antibiotic intraperitoneal therapy. At the clinical examination, the abdomen was painful with generalized muscular tenderness. Generalized peritonitis with false membranes (**Fig. 4**), thickened visceral and parietal peritoneum (**Fig. 5**) and inter-visceral adhesions were found intraoperatively. After a complete adhesiolysis, a careful exploration of the peritoneal cavity was made without finding any visceral lesions. 

In both cases, postoperative evolution was favorable, but peritoneal dialysis was stopped and the patients were transferred to hemodialysis.

The clinical presentation did not match the type of peritonitis in none of these cases. In the first case, poor symptomatology associated with mono-bacterial etiology, prompted the initial diagnosis of “catheter related” peritonitis, while in the second case, peritoneal irritation sings and intense pain suggested a secondary peritonitis. In fact, the correct diagnosis was completely the opposite. We considered these two cases representative for the difficulty of the differential diagnosis regarding the etiology of peritonitis associated with PD.

## Discussions

Peritonitis during PD has different features, which separate them from the “classical” surgical peritonitis [**3**].

The diagnosis of peritonitis during PD is established by the presence of at least 2 of the following:

- cloudy dialysis effluent with white blood cell count of more than 100 cells/ mm3 (usually more than 50% polymorphonuclear neutrophils) - present in 98% of the cases

- abdominal pain (present in approximately 75% of the cases)

- positive cultures from dialysate [**4**].

All the patients with cloudy effluent should be presumed to have peritonitis, which must be confirmed by effluent cell count, culture, and Gram staining [**5**]. The confirmation of bacterial or fungus presence is necessary because not all the instances of cloudy effluent reflect infectious peritonitis. Other causes that may be considered are chemical inflammation, hemoperitoneum, eosinophilia, malignancy, cloudy effluent taken from dry abdomen [**4**,**5**]. Biliary or fecal contamination of the dialysate strongly suggests secondary etiology of the peritonitis.

The presence of abdominal pain in a PD patient should include the peritonitis in the differential diagnosis even in the absence of the cloudy effluent [**4**]. The intensity of the pain in PD associated peritonitis varies from severe to mild or even absent. It is somehow organism specific (e.g. generally less with ConS, and greater with streptococcus, Gram-negative rods, S. aureus). Abdomen tenderness could be mild or even absent. Localized pain or tenderness should raise the suspicion of an underlying surgical pathology such as acute appendicitis or cholecystitis [**1**].

Other symptoms such as fever, nausea, or diarrhea are present in no more than half of all cases [**4**]. 

Treatment must be started empirically, prior to the knowledge of the causative organism. It consists in a broad spectrum antibiotics administrated mainly intraperitoneally. Intraperitoneal administration is superior to IV administration due to the possibility of achieving a higher concentration at the site and a lower toxicity [**1**]. The therapeutic recommendations of the International Society for Peritoneal Dialysis (ISPD) were first published in 1983 and revised five times, the last revision being made in 2010. With the appropriate treatment, a “catheter related” peritonitis resolution should be obtained in a few days.

The failure of the effluent to clear after 5 days of appropriate antibiotics defines a refractory peritonitis, which should be managed by removal of the catheter to protect the peritoneal membrane [**6**]. Other indications for catheter removal are refractory peritonitis, relapsing peritonitis, fungal peritonitis.

The challenge for the surgeon is the differential diagnosis of refractory and secondary peritonitis. Early surgical assessment and regular review of the case is necessary for distinguishing PD related peritonitis from surgical peritonitis under PD [**7**]. The dilemma must be solved quickly. A comprehensive clinical multidisciplinary assessment laboratory and imagery work-up for loculated collections, digestive and genital associated pathology should be undertaken. An incorrect diagnostic and a delayed etiological treatment may have severe consequences for the patient’s outcome. Exploratory laparotomy or laparoscopy should be considered regarding all patients with persistence of peritonitis signs after 5 days of correct antibiotic therapy [**3**].

## Conclusions

The differential diagnosis between a refractory PD peritonitis and a secondary surgical peritonitis under PD may be very difficult. Sometimes, only a meticulous intraoperative exploration can establish the correct etiological diagnosis. In front of a refractory PD peritonitis, surgical assessment must not be delayed. Also we have to keep in mind that the aim of the peritonitis treatment is the saving of the peritoneal membrane and not the contaminated catheter, which should be removed.

**Acknowledgment**

This paper is partly supported by the Sectorial Operational Programme Human Resources Development (SOPHRD), financed by the European Social Fund and the Romanian Government under the contract number POSDRU 141531.
